# *Staphylococcus aureus* Superantigen-Like Protein SSL1: A Toxic Protease

**DOI:** 10.3390/pathogens8010002

**Published:** 2019-01-01

**Authors:** Aihua Tang, Armando R. Caballero, Michael A. Bierdeman, Mary E. Marquart, Timothy J. Foster, Ian R. Monk, Richard J. O’Callaghan

**Affiliations:** 1 Department of Microbiology and Immunology, University of Mississippi Medical Center, Jackson, MS 39216, USA; acaballero@umc.edu (A.R.C.); mbierdeman@umc.edu (M.A.B.); mmarquart@umc.edu (M.E.M.); rocallaghan@umc.edu (R.J.O.); 2 Department of Microbiology, Moyne Institute of Preventive Medicine, Trinity College, Dublin 2, D02 PN40, Ireland; tfoster@tcd.ie (T.J.F.); ian.monk@unimelb.edu.au (I.R.M.); 3 Department of Microbiology and Immunology, Doherty Institute for Infection and Immunity, University of Melbourne, 3000 Melbourne, Australia

**Keywords:** *Staphylococcus aureus*, protease, superantigen-like protein, dimerization, cytokine

## Abstract

*Staphylococcus aureus* is a major cause of corneal infections that can cause reduced vision, even blindness. Secreted toxins cause tissue damage and inflammation resulting in scars that lead to vision loss. Identifying tissue damaging proteins is a prerequisite to limiting these harmful reactions. The present study characterized a previously unrecognized *S. aureus* toxin. This secreted toxin was purified from strain Newman *ΔhlaΔhlg*, the N-terminal sequence determined, the gene cloned, and the purified recombinant protein was tested in the rabbit cornea. The virulence of a toxin deletion mutant was compared to its parent and the mutant after gene restoration (rescue strain). The toxin (23 kDa) had an N-terminal sequence matching the Newman superantigen-like protein SSL1. An SSL1 homodimer (46 kDa) had proteolytic activity as demonstrated by zymography and cleavage of a synthetic substrate, collagens, and cytokines (IL-17A, IFN-γ, and IL-8); the protease was susceptible to serine protease inhibitors. As compared to the parent and rescue strains, the *ssl1* mutant had significantly reduced virulence, but not reduced bacterial growth, in vivo. The ocular isolates tested had the *ssl1* gene, with allele type 2 being the predominant type. SSL1 is a protease with corneal virulence and activity on host defense and structural proteins.

## 1. Introduction

*Staphylococcus aureus* causes infections in a wide variety of non-ocular sites and is a major ocular pathogen [1,2,3,4,5,6]. There are 27.6 cases of bacterial corneal infections per 100,000 people, and 130.4 cases per 100,000 contact lens users [7,8]. *S. aureus* is often described as the leading cause of corneal infections, including infections after LASIK surgery [4]. In a multi-center study, 42% of over 1,100 ocular *S. aureus* isolates were methicillin-resistant with a high concurrence of resistance to aminoglycosides, fluoroquinolones, and macrolides [8]. 

*S. aureus* secretes over thirty biologically active substances, of which many are virulence factors favoring bacterial survival in host tissue [9,10,11,12]. Two hemolytic toxins encoded by ≥ 98% of isolates [13,14], alpha-toxin (encoded by *hla*) and gamma-toxin (encoded by *hlg*), have been shown to contribute significantly to the pathology of experimental *S. aureus* keratitis [15,16]. Community- acquired MRSA strains may express another potential corneal virulence factor, Panton-Valentine leukocidin (PVL) [17]. In vitro, these secreted toxic proteins are produced late in the bacterial growth cycle due to the genetic regulation by the accessory gene regulatory (*agr*) system [18,19]. A key problem in keratitis is that virulence factors causing tissue damage lead to permanent scarring that decreases vision. An antibiotic that kills the infecting bacteria cannot inhibit the corneal damage mediated by bacterial toxins or enzymes. No chemical or immunological reagent is available for clinical use to stop the damage mediated by these bacterial products. 

The success of *S. aureus* as a tissue infecting pathogen is mediated by its ability to avoid host defenses. Among the immune evasion mechanisms are multiple superantigen-like proteins that inhibit components of both the adaptive and innate immune responses [12]. Staphylococcal superantigen-like proteins (SSL) share structural similarities with superantigens, but unlike superantigens, the SSL proteins do not bind MHC receptors or T cell receptors to elicit a toxic cytokine response [12]. SSL proteins have been shown to bind specifically targeted host defense proteins such as IgA, IgG, and complement components [20,21,22,23,24]. Koymans et al. demonstrated in vitro that SSL1 and SSL5 limit neutrophil chemotaxis and migration by inhibiting the activity of matrix metalloproteases, which provides a rationale for further studies to examine virulence of these proteins in an in vivo model of infection [25]. 

Alpha-toxin and, to a lesser extent, gamma-toxin are the potent corneal virulence factors produced by *S. aureus* [2]. *S. aureus* strain Newman was reported to lack alpha-toxin production and, since this toxin is important for corneal virulence, strain Newman was expected to have significantly reduced virulence relative to other *S. aureus* strains. However, when injected into the rabbit cornea, strain Newman produced a severe infection [15]. When the alpha-toxin and gamma-toxin genes were mutated to prevent any production of these two toxins, strain Newman retained an unexpected amount of virulence. The present study was initiated to identify a novel virulence factor that contributes to *S. aureus* corneal infections. The findings demonstrate that the superantigen-like protein SSL1 exhibits protease activity and plays an important role in virulence during a corneal infection. 

## 2. Results

### 2.1. Corneal Virulence of S. aureus Mutants

**Figure 1:**
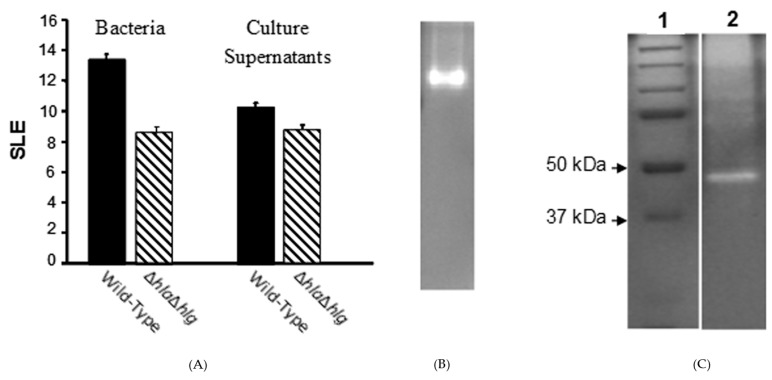
Ocular pathology of parental and double mutant *S. aureus* strain Newman and their culture supernatants in rabbit corneas and the protein contributing to virulence. (**A**) The SLE score at 24 h PI of *S. aureus* strain Newman double mutant (*ΔhlaΔhlg*; 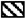 ) lacking alpha- and gamma-toxins, which produced substantial pathology as compared to normal eyes, was significantly less than that of the wild-type strain (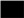 ) (*P* ≤ 0.001). When their culture supernatants were injected directly into the corneal stroma, the SLE score of the double mutant at 24 h after injection was substantial compared to normal eyes, but significantly lower than that of the wild-type strain (*P* = 0.004). (**B**) Non-denaturing (no SDS) zymogram of the pooled fractions of concentrated culture supernatant of the *S. aureus* double mutant (Newman *ΔhlaΔhlg*) containing toxic activities to the rabbit cornea. (**C**) A zymogram with SDS showing standard proteins (Lane 1) and the extracted toxin (Lane 2).

A rabbit model of experimental keratitis was used to determine virulence. Intra-corneal injection of 100 colony-forming units (CFU) of *S. aureus* strain Newman resulted in severe ocular pathology as measured by a slit lamp examination (SLE) score of 13.44 ± 0.43 at 24 h post-infection (PI). The Newman mutant deficient in both alpha- and gamma-toxins caused significantly less pathology than the wild-type strain (SLE score: 8.63 ± 0.35; *P* ≤ 0.001), but did mediate substantial pathology (Figure 1A) relative to normal eyes (SLE score: 0; data not shown). Both the wild-type and *ΔhlaΔhlg* strains grew to similar numbers of viable bacteria in the cornea (7.36 ± 0.06 and 7.30 ± 0.09 log CFU per cornea, respectively; *P* = 0.717).

### 2.2. Corneal Toxicity of Culture Supernatants

Intra-corneal injection of stationary phase culture supernatant of the wild-type or double mutant (*ΔhlaΔhlg*) resulted in SLE scores of 10.31 ± 0.40 or 8.75 ± 0.68, respectively (*P* = 0.004) (Figure 1A). Thus, despite lacking alpha- and gamma-toxins, the double mutant and its supernatant both demonstrated corneal virulence, implying an additional virulence factor was involved.

### 2.3. Identification of the Virulence Factor

**Figure 2:**
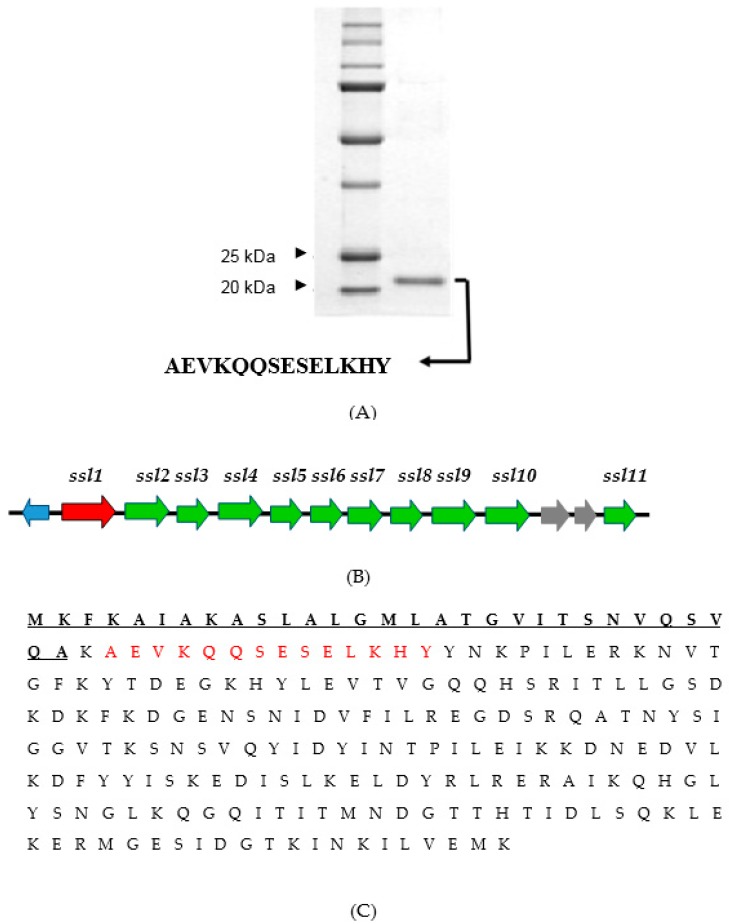
The virulence factor, SSL1, of *S. aureus* strain Newman. (**A**) SDS-PAGE of molecular weight standards and the isolated native protein under reducing and denaturing conditions showing a single band at about 23 kDa. The N-terminal sequence of the single band is shown below the gel. (**B**) The gene *ssl1* for the protease is the first gene located in pathogenicity island 2 (SaPln2). (**C**) The amino acid sequence of SSL1. The theoretical signal sequence is underlined. The amino acids obtained from N-terminal analysis are in red.

The concentrated supernatant of the double mutant was fractionated on a gel filtration column and then fractions were injected into rabbit corneas. Zymography of the fractions containing toxic activities under non-denaturing conditions demonstrated a single proteolytic band (Figure 1B). This protease extracted from the gel and, on a zymogram under denaturing conditions, was found to migrate at ~46 kDa (Figure 1C). This protease on a denatured and reduced SDS-PAGE migrated at ~23 kDa (Figure 2A), suggesting that dimerization was important for activity. The N-terminal sequence of 14 amino acids (i.e., A-E-V-K-Q-Q-S-E-S-E-L-K-H-Y) yielded in a BLAST search a 100% match with the SSL1 protein of strain Newman (from gene NWMN_0388; GenBank Accession Number BAF66660.1). Also, analysis of the SSL1 protein by mass spectrometry revealed only peptides matching the sequence of the Newman SSL1 protein (data not shown). The *ssl1* gene resides within the SaPIn2 pathogenicity island [12,26] (Figure 2B) and encodes for a 25.6 kDa protein containing a signal peptide. Cleavage of the signal sequence, as determined by ExPASy Signal P program, theoretically results in a secreted protein with a molecular weight of 22.6 kDa (Figure 2C), which agrees with the SDS-PAGE size determination of the isolated protein (Figure 2A). The *ssl1* gene, according to a BLAST search, is not present in other bacterial species, but is invariably found in all *S. aureus* strains examined, e.g., 88/88 sequenced genomes [26]. 

### 2.4. Recombinant SSL1: Immunogenicity and Toxicity

**Figure 3:**
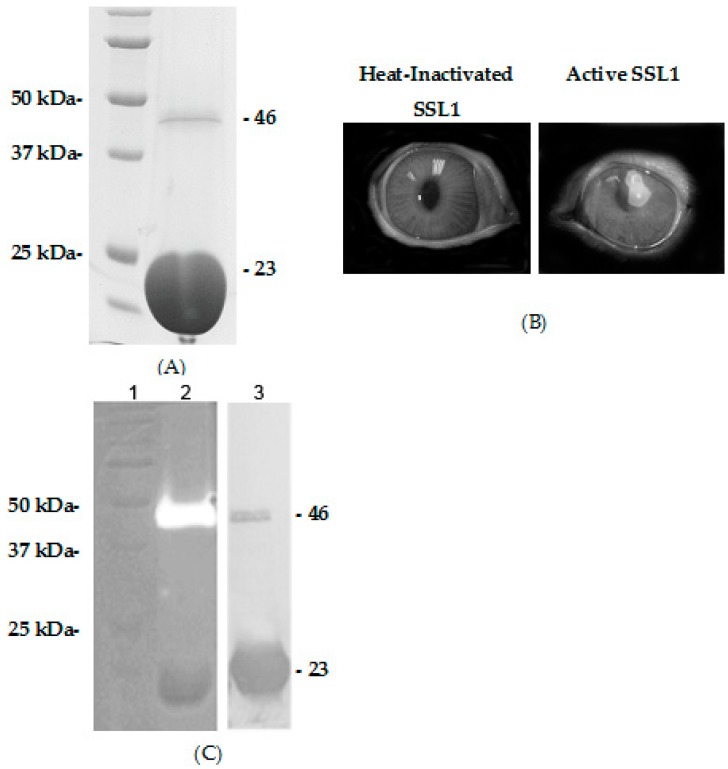
Recombinant SSL1 proteolytic activity and polymerization. (**A**) SDS-PAGE showing the standards (left lane), the dimer form of the recombinant protein at ~46 kDa, and the monomer at ~23 kDa (right lane). (**B**) Photographs of rabbit corneas injected with heat-inactivated (left photo) or active recombinant SSL1 (12 µg) at 24 h after injection (right photo). (**C**) Analysis of purified SSL1 including zymography (standards in Lane 1; recombinant SSL1 in Lane 2) and a Western blot of recombinant SSL1 in Lane 3. Please note that the protease activity is at the molecular size of a dimer (46 kDa), but not at the monomer size (23 kDa) and that both the monomer and dimer reacted with the antibody.

The *ssl1* gene was cloned and expressed in *E. coli* as a recombinant protein with a 6-His tag on the C-terminus. Recombinant SSL1 was purified from the culture supernatant by metal affinity chromatography followed by gel filtration (Figure 3A). The purified recombinant monomer fraction (~23 kDa) was used as an immunogen to induce the production of polyclonal antibody in rabbits. Corneas injected with active recombinant SSL1 (12 µg), but not the heat-inactivated recombinant protein, produced substantial pathology (Figure 3B). These pathological effects caused by the active recombinant SSL1 were also produced in eyes of rabbits immunized with the SSL1 monomer (Data not shown).

### 2.5. Recombinant SSL1: Polymerization

Recombinant SSL1 protein was purified from culture supernatant by affinity and gel filtration chromatography. SDS-PAGE of the recombinant protein showed a major band at ~23 kDa (monomer); however, some preparations also contained a dimer of ~46 kDa (Figure 3A). Zymography of the recombinant protein under denaturing conditions exhibited gelatinase activity at a molecular weight of ~46 kDa and, in some cases, a protease band at a higher molecular size (~80 kDa; data not shown), but not at 23 kDa (Figure 3C, Lane 2). The protein band migrating at ~46 kDa was also recognized on a Western blot by the polyclonal antibody raised against the SSL1 monomer (Figure 3C, Lane 3). This 46-kDa band was excised from the gel and analyzed by mass spectrometry confirming the identity as SSL1.

### 2.6. Recombinant SSL1: Enzymatic Properties

**Figure 4:**
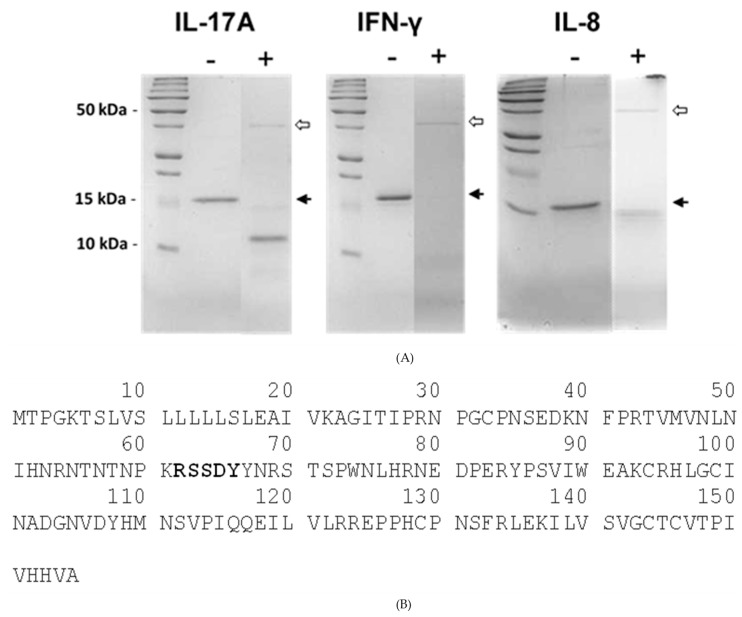
Degradation of host proteins by recombinant SSL1. (**A**) SDS-PAGE of human recombinant IL-17A (16 kDa), IFN-γ (17 kDa), or IL-8 (8.3 kDa) after incubation in phosphate buffer (-) or in the buffer plus active recombinant SSL1 (+; dimer: 100 ng) for two hours at 37 °C. Black arrows indicate the position of the un-cleaved substrate protein. White arrows indicate the position of the SSL1 dimer. (**B**) Amino acid sequence of human IL-17A. The 11-kDa breakdown product was sequenced by N-terminal analysis and the first five amino acids are RSSDY (shown in bold).

The active recombinant SSL1 of strain Newman was tested for proteolytic activity in vitro against several host proteins important to the immune response and was found to degrade IL-17A, IFN-γ, and IL-8 (Figure 4A). The 11-kDa breakdown product of IL-17A was N-terminally sequenced (Figure 4B) and, from this sequence, we conclude that SSL1 cleaves human IL-17A on the C-terminal side of a lysine (K61). The P_4_, P_3_, and P_2_ positions are threonine-58, asparagine-59, and proline-60, respectively.

**Figure 5:**
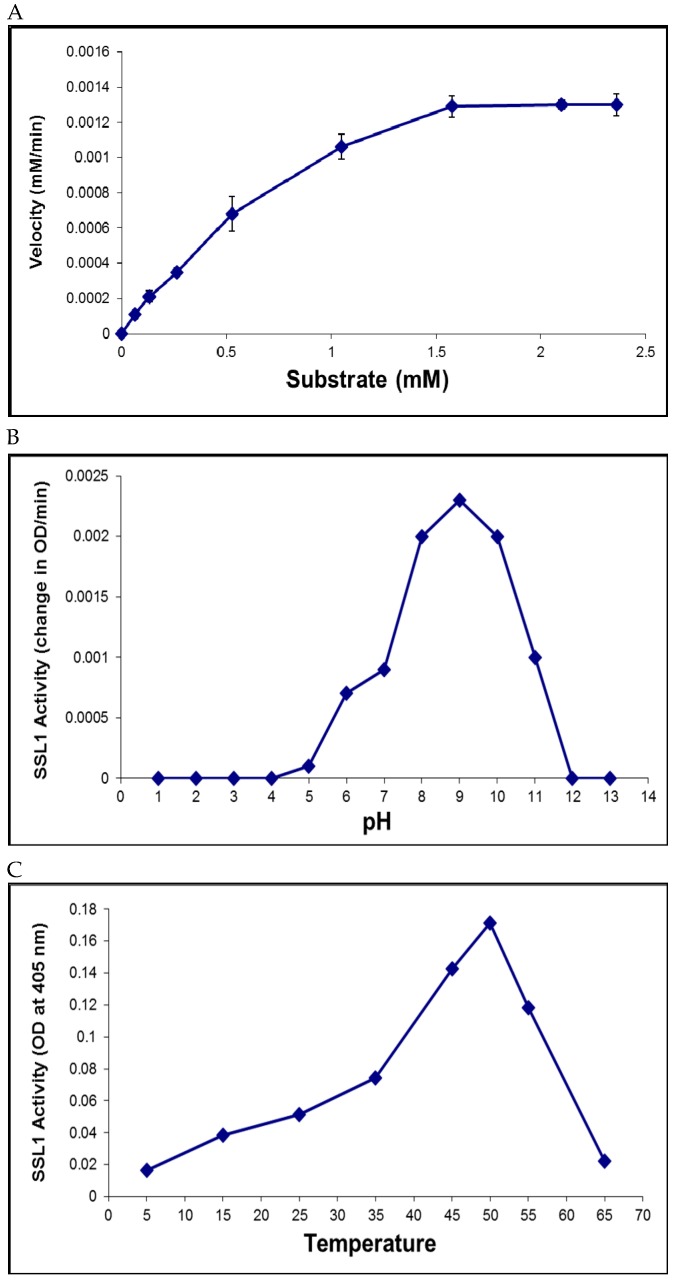
Enzymatic properties of SSL1. (**A**) Kinetic analysis of the hydrolysis of the substrate Chromozym PL. K_m_ (771 µM) and V_max_ (1.2 µM/min) were determined by incubating the enzyme with varying concentrations of the substrate ranging from 0 to 2.4 mM. (**B**) The optimal pH of SSL1 activity was determined using the CPL assay. The maximum protease activity was found at pH 9. (**C**) The optimal temperature for SSL1 activity was found to be 50 °C using the CPL assay.

A library of 29 chromogenic substrates was tested for cleavage by SSL1 with a focus on substrates ending with either a lysine or arginine. The substrate N-(p-Tosyl)-Gly-Pro-Lys-4 nitroanilide, known as Chromozym PL (CPL), was found to be susceptible and useful for assaying protease activity. Kinetic analysis of the hydrolysis of CPL determined that the K*_m_* and V*_max_* were 771 µM and 1.2 µM/min, respectively (Figure 5A). 

The effect of pH or temperature on SSL1 activity was also analyzed. The optimum pH for SSL1 activity was found to be pH 9, and the optimum temperature was 50 °C (Figure 5B,C). 

Eleven protease inhibitors were tested for their ability to block the hydrolysis of CPL by SSL1 (Table 1). The inhibitors demonstrating complete inhibition were AEBSF, TLCK, and chymostatin, all known to inhibit serine proteases. Partial inhibition (17 to 22%) was mediated by the metalloprotease inhibitor EDTA (10 mM) and the serine protease inhibitor aprotinin. 

**Figure 6:**
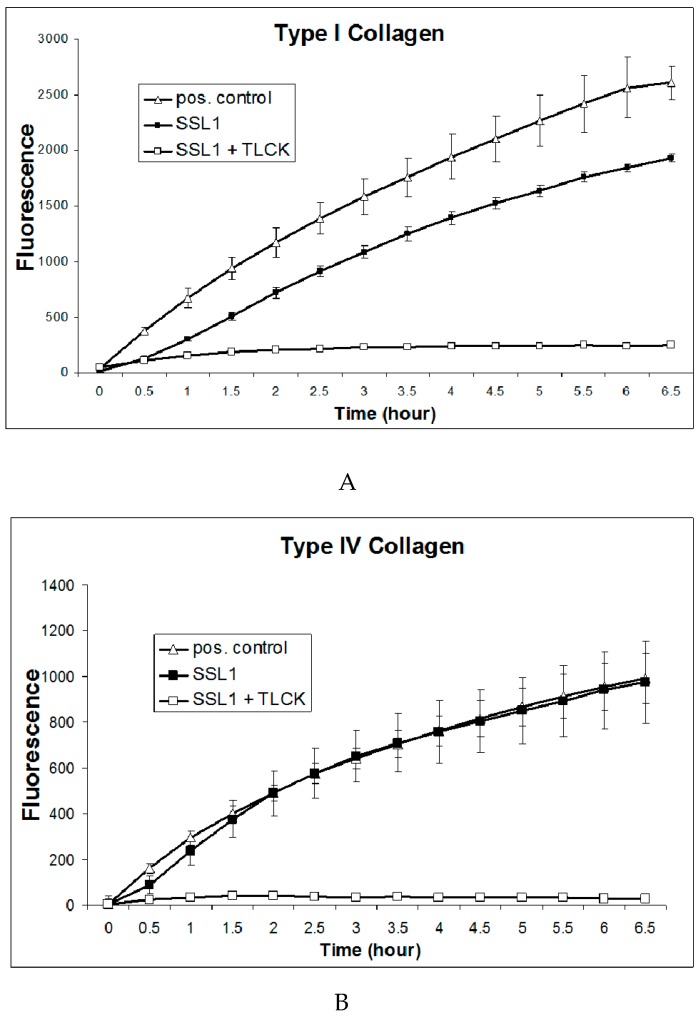
Cleavage of collagens by SSL1. (**A**) Recombinant SSL1 (20 µg), SSL1 plus TLCK (1 mM; negative control), or collagenase of *Clostridium histolyticum* (0.2 U/ml; positive control) was incubated with fluorescent collagen Type I (40 µg/ml) at 37 °C for 6.5 h in a microtiter plate reader and the change in fluorescence was measured every 30 minutes throughout the incubation. Background fluorescence, determined by no-enzyme reactions (blanks) was subtracted from each value. Data were expressed as the mean ± standard deviation. (**B**) SSL1 (20 µg), SSL1 plus TLCK (1 mM), or collagenase of *Clostridium histolyticum* (0.2 U/ml) was incubated with fluorescent collagen Type IV (40 µg/ml), as described in Figure 6A.

Furthermore, the recombinant SSL1 protease was found to cleave collagen type I and type IV (Figure 6A,B), which are structural components of the cornea [27,28]. 

### 2.7. Corneal Virulence of the SSL1 Deficient Mutant

**Figure 7:**
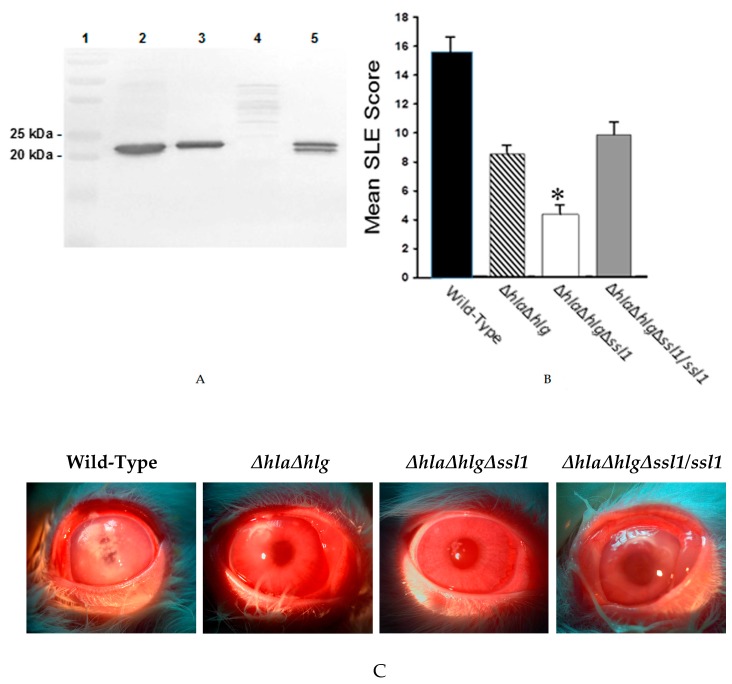
Phenotype confirmation and virulence of *S. aureus* strain Newman and the isogenic mutants. (**A**) Western blot analysis of concentrated culture supernatants of wild-type strain Newman and the mutants. Lane 1: molecular weight standards; Lane 2: strain Newman; Lane 3: double mutant (Newman *ΔhlaΔhlg*); Lane 4: triple mutant (Newman *ΔhlaΔhlgΔssl1*), and Lane 5: rescue strain (Newman *ΔhlaΔhlgΔssl1/ssl1*). The blot was developed using rabbit polyclonal antibody to recombinant SSL1. (**B**) Slit lamp examination scoring (SLE) of rabbit eyes 24 h after infection with strain Newman (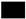 ), the double mutant (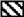 ), the triple mutant (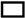 ), or the rescue strain (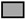 ). The SLE score for eyes infected with the triple mutant was significantly lower than that of eyes infected with the wild-type Newman, the double mutant, or the rescue strain (*, *P* ≤ 0.001). (**C**) Photographs of rabbit eyes 24 h after intrastromal injection with 100 CFU of strain Newman, the double mutant (Newman *ΔhlaΔhlg*), the triple mutant (Newman *ΔhlaΔhlgΔssl1*), or the rescue strain (Newman *ΔhlaΔhlgΔssl1/ssl1*). These eyes contained similar log CFU per cornea (*P* = 0.19).

To determine the role of SSL1 during *S. aureus* corneal infection, an isogenic mutant lacking the functional *ssl1* gene was constructed using the Newman *ΔhlaΔhlg* as the parental strain. This new mutant strain, Newman *ΔhlaΔhlgΔssl1*, a triple mutant, was complemented by inserting a functional *ssl1* gene (i.e., Newman *ΔhlaΔhlgΔssl1/ssl1*) at the native locus. The correct phenotype of these strains was confirmed by Western blotting of the concentrated culture supernatants using the antibody against recombinant SSL1 (Figure 7A). Virulence analysis of the Newman wild-type, *ΔhlaΔhlg* (the parent strain), the *ssl1*-deficient strain (*ΔhlaΔhlgΔssl1*), and the rescue strain demonstrated that the *ssl1*-deficient strain had less virulence (SLE score: 4.56 ± 0.49) than that of the wild-type strain, the parent strain (Newman *ΔhlaΔhlg*), and the rescue strain (SLE scores: 15.43 ± 1.10, 8.76 ± 0.59, and 10.82 ± 1.35, respectively; *P* ≤ 0.001) (Figure 7B). The *ssl1*-deficient strain lacked the virulence, especially corneal infiltrates, of the other strains (Figure 7B,C), yet grew in the cornea as well as the other strains (6.95 ± 0.07 log CFU for the *ssl1*-deficient strain vs 6.93 ± 0.14 log CFU for the wild-type, 6.77 ± 0.11 log CFU for the Newman *ΔhlaΔhlg* parent strain, and 6.90 ± 0.21 log CFU for the rescue strain; *P* = 0.19). 

### 2.8. ssl1 Alleles

There are 12 known alleles of the *ssl1* gene, with allele types 1, 2, and 3 being the most prevalent [26]. A set of clinical isolates including ones from ocular infections were analyzed to determine the presence and allele type of the *ssl1* gene. As shown in Table 2, the *ssl1* gene was found in all isolates tested (27/27) and six different allele types were identified with type 2 being the most prevalent among the ocular isolates (13/20). The amino acid sequence of SSL1 of MW2 (type 1 allele) shows 68.6% identity to that of Newman (type 3 allele) and the amino acid sequence of a type 2 ocular strain, strain 295.236, was 83.2% identical to that of strain Newman. The SSL1 protein of strain MW2 shares 78% identity with that of strain 295.236. 

**Figure 8:**
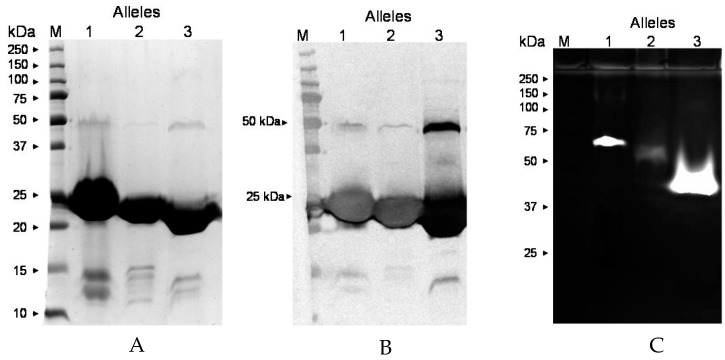
Properties of SSL1 proteins of *ssl1* alleles 1, 2, and 3. (**A**) SDS-PAGE of recombinant SSL1 proteins of alleles 1, 2, and 3 from strain MW2, 295.236, and Newman, respectively. Proteins were purified from culture supernatants by metal affinity and molecular sieve chromatography. The bands seen at approximately 23 kDa are the monomeric forms of the protein whereas the bands at approximately 46 kDa are the dimeric forms. M, molecular weight standards. (**B**) Western blot analysis of the proteins analyzed in Figure 8A using antibody to the SSL1 monomer of strain Newman (allele type 3). The bands seen at approximately 46 kDa are the dimers and the bands at approximately 23 kDa are the monomers. (**C**) Zymography (gelatin) of the proteins analyzed in Figure 8A. Despite having similar molecular weights, the proteolytic forms of recombinant proteins of alleles 1, 2, and 3 migrate differently, probably reflecting their differences in amino acid sequences.

To determine if protease activity is limited to only allele type 3, of which Newman is a member, the protease activity of the recombinant protein of the *ssl1* gene of strain MW2 (type 1), and ocular isolate 295.236 (type 2), was assayed. Figure 8A and 8B show the migration of the three different recombinant proteins on SDS-PAGE and a Western blot, respectively. Figure 8C shows that the recombinant SSL1 proteins of allele types 1, 2, and 3 have protease activity. 

## 3. Discussion

The present study demonstrates that SSL1 mediates ocular virulence and can proteolytically cleave host proteins, including two types of collagens and the human recombinant cytokines IL-8, IL-17A, and IFN-γ. The protease activity of SSL1 is a most unexpected finding because SSL proteins have been characterized to act by binding to and inhibiting molecules needed for multiple host functions including neutrophil migration, complement activity, blood clotting, wound healing, and antibody functions [12,19,20,21,22,23,24]. There has been no prior suggestion that any SSL protein has enzymatic activity; however, Fraser and Proft reported that dimer formation could be common among SSL proteins and that no function has been assigned to SSL dimers [12].

The significance of SSL1 is demonstrated by its corneal toxicity and the reduced virulence of the mutant lacking SSL1 production (Figure 7). SSL1 can cleave in vitro collagen types I and IV (Figure 6), both of which are structural components of the cornea [27,28]. Although SSL1 can cleave several proinflammatory cytokines (Figure 4) and is reported to inhibit the migration of neutrophils by inhibiting the matrix metalloproteases involved in neutrophil migration in vitro [25], overall the SSL1 protein causes an inflammatory reaction in the cornea. The inflammation could result from cleavage of protease activated receptors [29,30]. Corneal inflammation is known to contribute to the overall tissue damage and scarring of an infected cornea. The significance of SSL1 is also confirmed by the presence of the *ssl1* gene in all sequenced *S. aureus* genomes previously analyzed (88/88) [26]. The importance of SSL1 to corneal virulence was conclusively established by the significant reduction in virulence of the mutant lacking SSL1 production relative to the isogenic parent and rescue strains (Figure 7). 

SSL1 is a 226 amino acid protein sharing sequence homology with the other superantigen-like proteins. The structure, as derived from strain NCTC 8325, has recently been solved by Dutta et al. (available in the Protein Data Bank, ID#: 4O1N) [31]. The sequence of the *ssl1* gene of NCTC 8325 is identical to that of Newman indicating that the Newman SSL1, like that of NCTC 8325, has the beta-grasp domain at the C-terminus [31]. The beta-grasp domain has been reported to mediate dimerization of SSL7 and SSL11 by the internalization of hydrophobic residues allowing hydrogen bond formation by neutral residues of the two SSL monomers [23,32]. Al-Shangiti et al. suggested that SSL polymerization occurs adjacent to the external bacterial surface [32], a mechanism for SSL1 that could prevent its proteolytic activity within the bacterium. Because dimer formation is essential for SSL1 protease activity, such dimer formation could aid proteolysis by positioning together sequences distant from each other in the monomer.

SSL1 is a unique and novel protease sharing little sequence homology with any known protease. Searching the Pfam database, three hypothetical domains have been identified in SSL1 (Table 3), including a staphylococcal superantigen-like OB-fold domain, an MHC class II analogous protein (MAP) domain, and a staphylococcal/streptococcal toxin beta-grasp domain. No protease domain was found. Our inhibitor and host protein cleavage studies indicated that SSL1 could be a serine protease, employing the Asp-His-Ser triad in the active site. In the SSL1 sequence, there are 5 histidines, 15 aspartic acids, and 17 serines, so identifying the catalytic triad via site-directed mutagenesis will be time-consuming. Another complication in identifying the triad is that, whereas the structure of the SSL1 monomer has been determined [31], the structure of the dimer is unknown. To form a dimer, Al-Shangiti et al. suggested that the beta-grasp domain must undergo alterations in the location of multiple amino acid residues [33]. Apparently, since only the dimer is proteolytic, the available structural work on the monomer has been of limited value in identifying the triad. There is an example of another serine protease produced by *S. aureus* that undergoes a conformational change to produce a proteolytic triad. The exfoliative toxin is a serine protease whose active site is inactive until it undergoes a conformational change upon its interaction with a substrate protein [34].

The *ssl1* gene lies first in a series of ten contiguous superantigen-like genes in pathogenicity island two (SaPIn2) [12,26,35]. There are 12 known alleles of the *ssl1* gene with alleles 1-3 being the most prevalent [26]. In the present study, the *ssl1* gene was found in all of the 20 ocular isolates and 7 non-ocular isolates examined (Table 2); these *ssl1* genes were of six allelic types. Zymography revealed the proteolytic activity of the recombinant proteins of allele types 1, 2, and 3, despite the rather large differences (68.6% to 83.2% identity) in their sequences. These three proteins have similar but not identical molecular weights (22.622 kDa, 22.730 kDa, and 22.687 kDa, respectively) and they migrate at somewhat different rates on SDS-PAGE (Figure 8A). Their proteolytic forms also migrate differently on zymograms (Figure 8C), which is possibly due to the differences in their amino acid compositions. Additional allele types other than types 1-3, could also be proteolytic, but these have not yet been tested.

Numerous secreted proteins of *S. aureus* are regulated by the accessory gene regulator (*agr*) such that their production is limited to late log phase and stationary phase of growth [18]. The expression of the *ssl1* gene is up-regulated by two other regulatory systems, *rot* and *sae*, and down-regulated by *agr*, therefore SSL proteins are produced in the log phase, but not later in the growth cycle [10,36,37]. Benson et al. concluded that SSL proteins are active early in an infection [36]; thus, the ability of SSL1 to degrade pro-inflammatory cytokines could limit the initial host response to a developing infection. Naturally occurring *agr*-defective human isolates were found to over-produce SSL proteins that contribute to the virulence of the *agr*-defective strains [36]. 

Research to date shows that, when the cornea is infected with *S. aureus*, corneal pathology is mediated by at least three proven virulence factors; namely, alpha-toxin, gamma-toxin, and SSL1. For CA-MRSA strains, the corneal infection could involve a fourth virulence factor, Panton-Valentine leukocidin (PVL) [17]. Inhibition of the action of such aggressive molecules during keratitis would be an ideal means of limiting tissue damage while an antibiotic kills the infecting bacteria. Because SSL1 requires dimerization to achieve proteolytic activity, the application of an inhibitor of dimerization or of the protease activity could reduce the toxic effects of *Staphylococcus* keratitis.

## 4. Materials and Methods

### 4.1. Bacteria and Growth Conditions

*S. aureus* strains Newman, Newman *ΔhlaΔhlg*, Newman *ΔhlaΔhlgΔssl1*, Newman *ΔhlaΔhlgΔssl1/ssl1* were grown to equivalent optical density (OD_600_) at 37 °C for 24 h in tryptic soy broth or M9 minimal medium supplemented with 20 amino acids, casamino acids, and vitamins [15,16]. Antibiotics (10 µg/mL tetracycline and 10 µg/mL erythromycin) were added to the medium for strains with *hla* and *hlg* mutations. *E. coli* M15 [pREP4] with the expression vector pQE-60 was grown in LB medium with ampicillin 100 µg/mL and kanamycin 25 µg/mL. 

### 4.2. PAGE and Gelatin Zymography

Gelatin (0.1%) zymograms were performed using 10% acrylamide gels under non-denaturing conditions (no SDS) or denaturing conditions (with SDS) and stained with Coomassie blue [38]. Briefly, the gel containing gelatin was electrophoresed at 4 °C and 20 mA, then incubated in a reaction buffer consisting of 50 mM Tris-HCl (pH 8.0), 150 mM NaCl, 10 mM CaCl_2,_ and 1 µM ZnCl_2_ at 37 °C for 24 h. After staining with Coomassie blue, the area where proteolysis of gelatin occurred reveals clear (white) bands against a blue background.

### 4.3. Identification of S. aureus Virulence Factor

Culture supernatants were prepared, concentrated, and then fractionated by molecular sieve chromatography using Sephacryl S-100 [39]. Fractions (10 µL) were injected intrastromally into rabbit corneas and toxic fractions were analyzed by SDS-PAGE and zymography. Protein was extracted from the proteolytic band on zymogram and analyzed by SDS-PAGE. The band on SDS-PAGE was excised for N-terminal sequencing and mass spectrometry (University of Texas Medical Branch Biomolecular Resource Facility, Galveston, TX) [39,40].

### 4.4. Recombinant Protein Production

The gene was translated and the signal peptide cleavage site was identified using ExPASy’s software. For cloning into plasmid pQE-60, the NcoI and BglII restriction sites were incorporated into the PCR product. Genomic DNA of strain Newman *ΔhlaΔhlg*, ocular isolate 295.236, or strain MW2 was used as template in PCR reactions. The forward primer was 5′-AACCATGGGTAAATTTAAAGCGATAGCA-3′ and the reverse primer was 5′-TTAGATCTTTTCATTTCTACTAGAAT-3′. The recombinant protein was secreted by *E. coli*. All DNA constructs were verified by sequencing (MCLAB, South San Francisco, CA). The recombinant protein was purified from culture supernatants by metal affinity (TALON Resin; Clontech Laboratories, Mountain View, CA) and Sephacryl S-100 chromatography.

### 4.5. Production of Polyclonal Antibody 

Antibody to the recombinant Newman SSL1 protein monomer was produced in rabbits (*n* = 3) by methods described previously [16]. Briefly, rabbits were bled before immunization to acquire control sera (pre-bleed) and then they were injected subcutaneously with 100 µg SSL1 recombinant protein mixed 1:1 (vol/vol) with TiterMax Gold adjuvant (Sigma-Aldrich, St. Louis, MO). The immunization was repeated every 21 days for a total of three immunizations. For a negative control, rabbits were injected with adjuvant without the immunogen (i.e., mock immunized).

### 4.6. In Vitro Degradation of Host Proteins 

Recombinant Newman SSL1 (dimer, 0.1 µg) was incubated for 2 h at 37 °C with 1 µg of recombinant IL-17A, IFN-γ, or IL-8 (Applied Biological Materials Inc., Richmond, BC, Canada) and analyzed by SDS-PAGE. Recombinant SSL1 was also incubated with fluorescent collagen type I or type IV (Molecular Probes; Thermo Fisher Scientific, Waltham, MA) at 37 °C and the fluorescence (excitation at 485 nm and emission at 520 nm) was measured every 30 min for 6.5 h.

### 4.7. Enzyme Activity Assay, Kinetics, and Optimal Conditions 

Chromozym PL (CPL; Sigma-Aldrich) was used to determine SSL1 susceptibility to protease inhibitors. The substrate CPL mixed with enzyme samples was assayed in triplicate in a 96-well plate at 37 °C. Optical density (OD) at 405 nm was measured. Samples without enzyme or with heat-inactivated enzyme served as negative controls. The enzyme kinetics determination was performed by incubating SSL1 (20 µg) with varying concentrations of the substrate CPL at 37 °C and measuring OD_405_ in a microtiter plate reader every 10 minutes for 2 h. The K_m_ and V_max_ were determined by plotting the substrate concentration versus velocity using the GraphPad Prism 6 software. The optimum pH and temperature for activity were determined as described previously [39]. The optimal pH of SSL1 activity was determined by the CPL assay using potassium chloride - hydrochloric acid buffer (pH 1–2), citric acid - Na_2_HPO_4_ buffer (pH 3–7), Tris buffer (pH 8–9), sodium bicarbonate buffer (pH 10–11), or potassium chloride - sodium hydroxide buffer (pH 12–13). Briefly, the substrate was added to a reaction mixture of SSL1 (10 µg) and a buffer of a certain pH and OD_405_ was measured every 30 min for a 2.5-h period at room temperature. Controls consisted of the substrate at the pH tested without SSL1. To determine the optimal temperature for SSL1 activity, purified recombinant SSL1 (5 µg) was incubated with the substrate CPL at various temperatures ranging from 5 °C to 65 °C for 5 h. After incubation, OD_405_ was measured. Controls consisted of the substrate at the temperature tested without SSL1.

### 4.8. ssl1 Allele Typing of S. aureus Clinical Isolates 

The *ssl1* gene of the *S. aureus* isolates was amplified by PCR and sequenced in both directions (MCLAB, South San Francisco, CA). A pair of universal flanking primers was used: 5′-ATAGATTGGGGCTAAAAATT-3′ and 5′-ATAGCGCTTTGTTGTTAGAAAGTA-3′. PCR reactions were performed using 0.1 µg *S. aureus* genomic DNA, 50 picomoles of each primer, and the GoTaq green master mix from Promega (Madison, WI). The cycling conditions were as follows: 94 °C for 1 min, then 30 cycles of 94 °C for 20 s, 54 °C for 20 s, and 68 °C for 45 s. The resulting *ssl1* gene sequences were compared to the allele type sequences as described by McCarthy and Lindsay [26]. 

### 4.9. Construction of Isogenic Mutants 

The Newman *ΔhlaΔhlg* double mutant (Ery^R^/Tet^R^) was created by phage transduction of the antibiotic markers from *S. aureus* strain 8325-4 *ΔhlaΔhlg* with phage 85. To create the triple mutant (Newman *ΔhlaΔhlgΔssl1*), the *ssl1* gene was deleted from the chromosome of the double mutant by allelic exchange without an antibiotic marker. A construct for the unmarked deletion of *ssl1* was made by SOE-PCR [41]. An upstream AB fragment 

(A primer 5′-TATATCTAGAATATTCATCAATTCTGGTAAATATGG-3′ XbaI; B primer 5′-CATAATTTTTAGCCCCAATCTATTTAAATTTG-3′) and downstream CD fragment (C primer 5′-AGATTGGGGCTAAAAATTATGTAATACTTTCTAACAACAAAGCGCTATG-3′; D primer 5′-ATATCTGCAGTACGGTCTTCTTGTAACTTTTTATCC-3′ Pstl) were amplified and joined by PCR using Phusion polymerase (New England Biolabs, Ipswich, MA). The product was digested with XbaI/PstI and ligated into pIMC5 digested with SpeI/PstI and transformed into *E. coli* DH10B. The 2-kb insert was verified by sequencing. The construct pIMC5-*ssl1* was transformed into *S. aureus* RN4220 and then transduced into Newman *ΔhlaΔhlg* with phage 85. Allelic exchange was conducted as described previously [41] with potential Newman *ΔhlaΔhlgΔssl1* mutants screened with OUT primers (5′-TAATTAATACGACCATCGCCAGC-3′ / 5′-TCGTCATCAAGTATATGTGTTATGC-3′). To restore the *ssl1* gene in the Newman *ΔhlaΔhlgΔssl1* background, the *ssl1* gene and flanking sequence were amplified from Newman genomic DNA with the above A and D primers. The product was cloned into pIMC5 producing pIMC5*ssl1*^+^ and allelic exchange performed in the Newman *ΔhlaΔhlgΔssl1* background yielding Newman *ΔhlaΔhlgΔssl1/ssl1*.

### 4.10. Corneal Toxicity Model 

The use of animals, New Zealand white rabbits (2.0–3.0 kg) of either sex, adhered to the protocol (1060D) approved by the Institutional Animal Care and Use Committee of the University of Mississippi Medical Center and the Association for Research in Vision and Ophthalmology Statement for the Use of Animals in Ophthalmic and Vision Research. Rabbits were anesthetized by subcutaneous injection of a mixture of xylazine (100 mg/mL; Butler Co., Columbus, OH) and ketamine (100 mg/mL; Fort Dodge Animal Health, Fort Dodge, IA). Proparacaine (Bausch and Lomb, Tampa, FL) was topically applied to the eyes prior to intrastromal corneal injections (10 µL) of recombinant toxin, concentrated supernatant (50-fold), or 100 CFU of *S. aureus* in 10 µL tryptic soy broth (*n* ≥ 10 eyes/group) [16]. 

### 4.11. Slit Lamp Examination Scoring and Quantification of Viable Bacteria 

Rabbit eyes underwent slit lamp examination scoring (SLE) as described by Arana et al. [42]. Briefly, SLE scoring involved two masked observers grading on a scale of 0 to 4 for each of seven ocular parameters (conjunctival injection, conjunctival chemosis, corneal edema, corneal infiltration, iritis, fibrin accumulation in the anterior chamber, and hypopyon formation). The parameter scores were added to yield a SLE score that ranged from 0 for a normal eye to a theoretical maximal score of 28 for a highly inflamed and damaged eye. SLE scores were not allowed to go to or above 20. Log CFU per cornea was determined as previously described [16]. Briefly, rabbit corneas were harvested at 24 h PI and homogenized in 3.0 mL sterile phosphate-buffered saline (PBS). A 0.1-ml aliquot of the homogenate was serially diluted 1:10 in PBS and plated on tryptic soy agar (TSA) in triplicate. Plates were incubated at 37 °C for 24 h. Colonies were counted and expressed as base 10 logarithms.

### 4.12. Statistical Analysis 

Mean and standard error of the mean (SEM) were calculated using Microsoft Excel. Statistical analyses of SLE scores and CFU determinations were performed using SAS software [15]. For SLE scores, statistical analyses of inter-group differences were performed using non-parametric one-way analysis of variance (Kruskal-Wallis test). For CFU determinations, one-way analysis of variance (ANOVA) and Student’s *t* tests between least-squared means from each group were performed. *P* ≤ 0.05 was considered significant. 

## Figures and Tables

**Table 1:** Inhibitors tested for SSL1 enzymatic activity using CPL assay.

Inhibitor	Target Protease Class	Concentration Used	% Activity of Control ^1^
None	-	-	100
Chymostatin	Cys/Ser	0.1 mM	0
AEBSF	Serine	10 mM	0
TLCK	Serine	1 mM	0
EDTA	Metallo	10 mM	78
Aprotinin	Serine	0.001 mM	83
Antipain	Cys/Ser	0.1 mM	100
Pepstatin	Aspartic	0.001 mM	101
Leupeptin	Cys/Ser	0.1 mM	110
Bestatin	Metallo	0.1 mM	119
E-64	Cysteine	0.01 mM	130
Phosphoramidon	Metallo	1 mM	137

^1^ These values indicate the changes of SSL1 activity in the presence of an inhibitor relative to the inhibitor-free control using the Chromozym PL assay.

**Table 2:** The *ssl1* allele types of *Staphylococcus aureus* clinical isolates.

Ocular Isolates			
Strain	Allele Type ^1^	Strain	Allele Type ^1^
05.4144 ^†^	2	82936 ^§^	2
06.6451 ^†^	1	83523 ^§^	2
11.14697 ^†^	5	85278 ^§^	2
13.9793 ^†^	3	85397 ^§^	2
190.164 ^‡^	7	91731 ^§^	2
279.274 ^‡^	3	SD22125 ^§^	3
295.236 ^‡^	2	CO19432 ^§^	1
43506 ^§^	2	CO61879 ^§^	2
67993 ^§^	2	CO63355 ^§^	2
70468 ^§^	2	CO63825 ^§^	2
**Non-Ocular Isolates**			
**Strain**	**Allele Type ^1^**	**Strain**	**Allele Type ^1^**
MRSA 301 ^||^	3	76.137 ^‡^	2
MRSA 306 ^||^	3	103.93 ^‡^	3
44.255 ^‡^	1	116.60 ^‡^	9
63.21 ^‡^	2		

^1^ Genomic DNA of *S. aureus* clinical isolates was used as template for PCR reactions to amplify the *ssl1* gene. The PCR product was sequenced and compared to the previously published *ssl1* alleles [26]. † These strains were provided by Eric G. Romanowski and Regis P. Kowalski of the Campbell Laboratory of the University of Pittsburgh. ‡ These strains were isolated at the University of Mississippi Medical Center. § These strains were provided by Dr. David Stroman, Alcon Laboratories, Inc. || These strains were isolated at Louisiana State University Health Sciences Center.

**Table 3:** Bioinformatic analysis of SSL1 domains (motifs) using the Pfam database.

Motif ID ^1^	Amino Acids	Definition	*E*-Value
pf: SSL_OB	41–123	Staphylococcal superantigen-like OB-fold domain	4.9 × 10^−32^
pf: MAP	142–209	MAP domain	0.014
pf: Stap_Strp_tox_C	147–206	Staphylococcal/Streptococcal toxin, beta-grasp domain	0.0002

^1^ Motif search was performed using the Pfam database (available in the public domain at https://www.genome.jp/tools/motif/).
